# Multi-electrolyte disturbance and supplementation in severely malnourished hospitalized adolescents with restrictive eating disorders

**DOI:** 10.1186/s40337-023-00919-4

**Published:** 2023-11-15

**Authors:** Eva-Molly Petitto Dunbar, Chase Pribble, Jennifer Cueto, Andrea B. Goldschmidt, Christina Tortolani, Abigail A. Donaldson

**Affiliations:** 1 University of California San Diego Eating Disorders Center for Treatment and Research, San Diego, CA 92121 USA; 2 Warren Alpert Medical School of Brown University, 222 Richmond St., Providence, RI 02903 USA; 3 Rhode Island Hospital, 593 Eddy St., Providence, RI 02903 USA; 4 University of Pittsburgh School of Medicine, 3711 O’Hara St., Pittsburgh, PA 15213 USA; 5 Rhode Island College, 600 Mount Pleasant Ave., Providence, RI 02908 USA

**Keywords:** Restrictive eating disorders, Adolescents and young adults, Refeeding, Hospitalization

## Abstract

**Background:**

This study describes the prevalence of hypophosphatemia, hypokalemia, and/or hypomagnesemia and resulting electrolyte supplementation during refeeding in severely malnourished youths hospitalized for restrictive eating disorders.

**Methods:**

Hospitalized patients between 11-26y (*N* = 81) at < 75% treatment goal weight (TGW) were assessed through retrospective chart review. Outcomes were compared between participants < 70% TGW and those 70–75% TGW. Nutritional rehabilitation started at 1750 kcals/day and advanced by 500 kcal every other day until target intake was achieved. Associations between %TGW on admission; hypophosphatemia, hypokalemia, and/or hypomagnesemia; and electrolyte supplementation were examined.

**Results:**

Of the 24 (29.6%) participants with hypophosphatemia, hypokalemia, and/or hypomagnesemia, 7 (8.6%) received supplementation; the remainder corrected without supplementation. Participants < 70% TGW did not differ from those 70–75% TGW on rates of these conditions or need for supplementation.

**Conclusions:**

Hospital-based nutritional rehabilitation did not confer increased rates of hypophosphatemia, hypokalemia, and/or hypomagnesemia or need for electrolyte supplementation in patients < 70% TGW compared to those 70–75% TGW. While additional research is needed to establish clinical practice guidelines on electrolyte management in this population, our findings suggest that nutritional rehabilitation may be reasonably undertaken without prophylactic electrolyte supplementation, even in patients < 70% TGW.

## Abstract (plain language summary)

Identifying safe management methods for nutritional rehabilitation among severely malnourished hospitalized adolescents and young adults with restrictive eating disorders can expedite discharge planning and improve treatment outcomes. In contrast to past studies on inpatient nutritional rehabilitation, this study describes the prevalence and management of multiple-electrolyte disturbance (hypophosphatemia, hypokalemia, and/or hypomagnesemia) during refeeding among hospitalized severely malnourished youths with restrictive eating disorders. We found that in this population, with experienced, close medical supervision, nutritional rehabilitation may be reasonably undertaken without prophylactic electrolyte supplementation, even in patients < 70% goal treatment weight. These findings can inform hospital-based refeeding protocols for adolescents and young adults with restrictive eating disorders, where the practice around prophylactic supplementation is variable.

## Introduction

Early, aggressive nutritional intervention among hospitalized patients with restrictive eating disorders (RED) is safe, shortens hospitalization, and improves treatment response [1–9]. Renourishment is key to addressing medical and emotional complications of RED, a process critical to recovery [10–12].

Refeeding syndrome (RS)—a potentially life-threatening metabolic response to abrupt nutritional advance involving hypophosphatemia, hypokalemia, and hypomagnesemia—is one risk of aggressive nutritional rehabilitation [13]. RS is relatively uncommon in hospital settings due to preventive practices (e.g., gradually advancing nutrition while monitoring serial electrolytes/exam findings) [11, 14], most often occurring among individuals < 70% of treatment goal weight (TGW) [10, 15] in the first week of nutritional rehabilitation [2, 13, 16]. Most studies examining RS focus only on hypophosphatemia without considering associated hypokalemia and hypomagnesemia [15], or appreciating normal physiologic variability and other potential causes of electrolyte disturbance in malnourished individuals [13]. The current study examined changes in multiple electrolytes and need for supplementation in hospitalized youths < 70% TGW undergoing refeeding; we expected low rates of RS and minimal need for supplementation.

## Methods

### Participants and procedures

We studied inpatients < 75% TGW (ages 11–26y) medically hospitalized with RED between May, 2015–February, 2021. The target weight range was selected because it aligns with the criteria for medical hospitalization from the Society for Adolescent Health and Medicine and allows for comparison of severely and moderately malnourished inpatients [10, 11, 15]. Participants were identified through electronic medical record (EMR) reports of inpatients admitted to the Adolescent Medicine eating disorder service at < 75% TGW on admission. Participants were managed on a standard refeeding protocol including a daily multivitamin but no targeted prophylactic electrolyte supplementation for RS; hospital formulary multivitamins used during the study period did not contain any phosphorus, potassium, or magnesium. Twelve participants were admitted more than once during the study period; each admission was considered a separate encounter. Participants were categorized as moderately (< 75% TGW) or severely (< 70% TGW) malnourished on admission. Inpatient dietitians used a standard approach to estimating an individual’s TGW, including consideration of both percent of the mean BMI for age and change from pre-morbid BMI growth trajectory [11]. Hospitalization criteria aligned with established clinical guidelines [11].

Phosphorus, potassium, and magnesium were drawn every morning for 5 days following initiation of the protocol and on alternating days thereafter; additional laboratory testing was obtained as clinically indicated. Potassium ≤ 3.5 mEq/L; phosphorous ≤ 3.0 mg/L; and/or magnesium ≤ 1.4 mEq/L were considered abnormally low. Participants typically started a 1750 kcals/day diet and advanced by 500 kcal every other day until target intake was achieved; exceptions were directed by the team’s clinical judgement (e.g., younger patients with lower caloric goals might start below 1750 kcal/day). Since electrolyte disturbance in malnutrition can have various etiologies which inform management, the decision to replete electrolytes was made based on clinical assessment of laboratory findings (results, trend over time, and single vs multiple electrolyte disturbance), vitals, and physical exam. The study was approved by the hospital’s Institutional Review Board.

### Measures

Retrospective chart review identified demographics; anthropometrics; reason for hospitalization; illness severity; nutritional plan; laboratory findings; and electrolyte interventions to address RS (Table 1). Two researchers cross-checked 20% of the data.

**Table 1:** Demographics and clinical data

	Inpatients at 70–75% TGW (N = 52 unless stated otherwise)				Inpatients at < 70% TGW (N = 29 unless stated otherwise)				*P value*
	N	Percent	Range	Mean (± SD)	N	Percent	Range	Mean (± SD)	
*Gender*									
Female	46	88.5%			27	93.1%			
Male	5	9.6%			2	6.9%			
Transgender	1	1.9%							
*Race*									
Caucasian	47	90.4%			23	79.3%			
Asian	3	5.8%			1	3.4%			
Not specified	2	3.8%			5	17.2%			
*Ethnicity*									
Non-Hispanic/Latino	51	98.1%			24	82.8%			
Hispanic/Latino	1	1.9%			4	13.8%			
Not specified					1	3.4%			
*Diagnosis*									
Anorexia nervosa	31	59.6%			18	62.1%			
Avoidant-restrictive									
Food intake disorder	2	3.8%			2	6.9%			
Unspecified eating disorder	19	36.5%			9	31.0%			
Age (years)			11.6–25.2	17.8 (3.5)			12.4–24.6	17.0 (3.5)	0.327
Height (cm)			140–180	162.5 (8.8)			143–180	161.1 (11.0)	0.533
Admission weight (kg)			25–60.6	41.0 (5.9)			23.9–44.9	36.0 (5.8)	< 0.001*
Admission TGW (kg)			34.6–82.6	56.7 (8.1)			35.3–70.2	55.0 (9.0)	0.381
%TGW on admission			70–74%	72.3% (1.3%)			55–69%	65.6% (3.2%)	< 0.001*
Admission BMI (kg/m^2^)			12.6–19.7	15.5 (1.3)			11.3–17.1	13.8 (1.3)	< 0.001*
BMI z-score			− 4.9 to (− 0.15)	− 2.6 (1.2)			− 7.8 to (− 2)	− 3.8 (1.6)	< 0.001*
Percent of total weight lost since onset of restrictive behaviors	50		3.6–55%	23.8 (10.5)			6–40.5%	27.0 (9.3)	0.178
Duration of illness (months)	51		1–150	20.0 (33.5)			3–36	15.1 (9.9)	0.445
Amenorrhea (months)	23		2–27	7.2 (7.6)	16		0–24	7.8 (8.2)	0.816
Caloric intake prior to admission (kcal/day)	38		450–3500	1446 (757)	24		300–3500	1322 (706)	0.522
Initial inpatient caloric intake (kcal/day)			1250–4250	1856 (685)			1000–4000	1741 (621)	0.456
Caloric increase (kcal/day)			0–500	239 (79)			0–333.33	249 (62)	0.559
Length of admission (days)			2–17	7.8 (3.1)			2–24	11.2 (4.9)	< 0.001*
*Electrolyte derangements*	14	26.9%			10	34.5%			
Potassium ≤ 3.5 mEq/L	9	64.2%			7	70.0%			
Phosphorous ≤ 3.0 mg/L	8	57.1%			6	60.0%			
Magnesium ≤ 1.4 mEq/L	0	0%			1	10.0%			
Received electrolyte supplementation	4	7.6%			3	10.3%			
Discharge caloric intake (kcal/day)			1750–4250	3370 (455)			2000–4250	3500 (551)	0.257
Discharge weight (kg)			27–62.9	43.7 (6.2)			27.2–52.1	40.0 (6.3)	0.012
Discharge %TGW			69–83%	77.1% (3.2%)			61–80%	72.9% (3.7%)	< 0.001*
Overall change in percent of TGW			− 1 to 10%	4.8% (2.9%)			− 1 to 12%	7.3% (3.3%)	< 0.001*
Discharge BMI			13.3–20.5	16.5 (1.5)			12.9–18.46	15.3 (1.4)	< 0.001*
Overall change in BMI			− 0.2 to 2.3	1.0 (0.6)			0–2.70	1.5 (0.7)	0.001

*p < *0.001*

### Statistical analyses

Descriptive statistics were applied to demographic and nutrition-related variables. Independent t- and chi-square tests compared participants < 70% and 70–75% TGW on rates of hypophosphatemia, hypokalemia, and/or hypomagnesemia, and supplementation receipt.

## Results

Among 81 encounters, %TGW on admission ranged from 55 to 74% (69.8% ± 4.0%), with 35.8% admitted at < 70% TGW. Of those < 70% TGW, 97% were > 60% TGW. Demographics were similar between < 70% and 70–75% TGW groups (Table 1). Comorbid psychiatric conditions were present in 79.3% of participants (anxiety: 94%; mood: 56%; developmental disorders: 12%). Medical co-morbidities included gastrointestinal (19.0%), pulmonary (9.5%), neurologic (7.8%), endocrine (6.3%), hematologic (4.8%), and cardiac (3.2%) concerns.

Though ranges of caloric start and advance were wide, nearly ~ 80% of participants both started between 1500–2500 kcal/day and increased by 250–350 kcal/day. There were no differences between those 70–75% versus < 70% TGW in pre-admission caloric intake, initial inpatient caloric requirements, average daily increases, or discharge caloric requirements (Table 2). The two groups differed on length of stay and change in %TGW during admission (*p* < 0.05).

**Table 2:** Analysis of subgroups

Variable	Inpatients at 70–75% TGW				Inpatients at < 70% TGW			
	Electrolyte derangements°		Electrolyte supplementation		Electrolyte derangements°		Electrolyte supplementation	
	*t*	Sig.	*t*	Sig.	*t*	Sig.	*t*	Sig.
Percent of TGW	1.683	0.099	3.553	0.012	3.046	0.005*	3.784	< 0.001*
BMI z-score^	1.972	0.056	0.492	0.626	1.932	0.067	2.670	0.014
Caloric intake on admission	1.305	0.038	1.194	0.240	1.812	0.067	− 0.324	0.749
Initial inpatient caloric intake	1.136	0.261	0.698	0.488	1.760	0.090	1.416	0.168
Advancing by	0.275	0.785	− 0.284	0.777	− 0.216	0.831	1.468	0.154
% total weight lost	1.134	0.263	0.414	0.681	− 2.301	0.029	− 2.105	0.045
Duration of Illness	− 1.340	0.204	− 1.430	0.246	− 0.746	0.462	− 2.643	0.014

*p < *0.01*

°Electrolyte derangement defined as: potassium ≤ 3.5 mEq/L; phosphorus ≤ 3.0 mg/L; magnesium ≤ 1.4 mEq/L

^BMI z-score was only determined in participants ≤ 20 years old (N = 16) due to lack of standardized percentiles above this age

Hypophosphatemia, hypokalemia, and/or hypomagnesemia were present in 26.9% of participants 70–75% TGW and 34.5% of participants < 70% TGW (29.6% of the entire sample). Supplementation was prescribed in 22.5% of cases where levels were low (8.6% of the entire sample): 5 received phosphorous and potassium; 1 potassium; 1 phosphorous; none received magnesium. Nadirs triggering supplementation ranged from 2.4–3.3 mEq/L for potassium and 2.0–2.8 mg/L for phosphorus. Average nadirs among participants receiving the intervention were: phosphorous 2.4, magnesium 1.4, and potassium 3.0. Participants who did not receive supplementation were monitored closely and experienced physiologic correction. Among participants who did not receive supplementation, average nadirs were as follows: phosphorous 2.8, potassium 3.4, and there were no magnesium abnormalities in this group. Rates of electrolyte abnormalities and supplementation did not differ between the two groups (Chi-squared 0.51, *p* = 0.475; 0.17, *p* = 0.684 respectively).

Seven variables were tested for association with electrolyte disturbance and/or need for supplementation (Table 2). Percent TGW was associated with both outcomes only in participants < 70% TGW (3.046, 3.784, *p* < 0.01).

## Discussion

This study examined electrolytes associated with RS and found that rates of hypophosphatemia, hypokalemia, and/or hypomagnesemia were low despite the absence of prophylactic electrolyte supplementation. Though supplementation was more common among participants < 70% TGW, findings suggest that even in these lowest-weight patients, assertive nutritional advancement may be safely undertaken in the inpatient setting with vigilant watchful waiting and informed decision-making regarding electrolyte monitoring and management.

Standard practice for electrolyte management in early refeeding of hospitalized patients with RED has not been established [15]. Concern for RS typically arises based on review of an electrolyte panel alongside physical assessment [13, 15]. Universal supplementation prevents clear assessment of an individual’s physiologic function in a time of metabolic transition. Indeed, the majority of study patients with electrolyte derangements corrected without intervention, which suggests that watchful waiting—as opposed to reflexive or prophylactic supplementation—may be appropriate for hospitalized patients with severe malnutrition.

Our study had several limitations. First, the sample size was small, presenting a challenge to data analysis by smaller sub-groups (e.g. age), though our focus on severely malnourished inpatients addresses a notable gap in the existing literature. Second, although dietary management was generally consistent with an established protocol, data were collected over several years during which the protocol’s caloric starting point and advance were increased to reflect adjustments in standards of care [1]; this prevented application of universal caloric metrics. Similarly, supplementation was not implemented systematically or in a randomized fashion due to pragmatics in the clinical context (i.e., multiple factors needing to be considered/further assessed to determine supplementation), thus outcomes could not be definitively linked to supplementation status. Third, sociodemographic descriptors were limited by data in the EMR, including binary gender terms and broad characterization of race/ethnicity. Fourth, several participants were admitted multiple times, which may have introduced confounds related to severity or duration of illness; however, repeat admissions are part of the reality of inpatient eating disorder care and therefore important to consider in examination of RS in this setting. Finally, hypophosphatemia, hypokalemia, and/or hypomagnesemia in RED cannot always be attributed to RS; other potential reasons for electrolyte disturbance (e.g., purging) might present confounding factors.

Inpatient providers routinely contend with refeeding risks without a clear definition of RS [17] or standard electrolyte management guidelines. By comprehensively examining electrolytes during refeeding without use of prophylactic supplementation, this study might contribute to future guidelines on how to best manage RS risk in vulnerable individuals.

## Data Availability

The data that support the findings of this study are available from the corresponding author upon reasonable request (epetittodunbar@uri.edu).

## Abbreviations

RED: Restrictive eating disorder
TGW: Treatment goal weight
RS: Refeeding syndrome
BMI: Body mass index
EMR: Electronic medical record
